# A comparison of the bilateral decompression via unilateral approach versus conventional approach transforaminal lumbar interbody fusion for the treatment of lumbar degenerative disc disease in the elderly

**DOI:** 10.1186/s12891-021-04026-w

**Published:** 2021-02-08

**Authors:** Yifan Huang, Jian Chen, Peng Gao, Changjiang Gu, Jin Fan, Zhiyi Hu, Xiaojian Cao, Guoyong Yin, Wei Zhou

**Affiliations:** Department of Orthopedics, The First Affiliated Hospital of Nanjing Medical University, No. 300 Guangzhou Road, Nanjing, 210029 People’s Republic of China

**Keywords:** Bilateral decompression via unilateral approach, Lumbar degenerative disease, Fatty degeneration

## Abstract

**Background:**

Bilateral decompression via unilateral approach (BDUA) is an effective surgical approach for treating lumbar degenerative diseases. However, no studies of prognosis, especially the recovery of the soft tissue, have reported using BDUA in an elderly population. The aims of these research were to investigate the early efficacy of the bilateral decompression via unilateral approach versus conventional approach transforaminal lumbar interbody fusion (TLIF) for the treatment of lumbar degenerative disc disease in the patients over 65 years of age, especially in the perioperative factors and the recovery of the soft tissue.

**Methods:**

The clinical data from 61 aging patients with lumbar degenerative disease who received surgical treatment were retrospectively analyzed. 31 cases who received the lumbar interbody fusion surgery with bilateral decompression via unilateral approach (BDUA) were compared with 30 cases who received conventional approach transforaminal lumbar interbody fusion. The radiographic parameters were measured using X-ray including lumbar lordosis angle and fusion rate. Japanese Orthopedic Association (JOA), Visual Analogue Scale (VAS) and Oswestry Disability Index (ODI) scores were used to evaluate the clinical outcomes at different time points. Fatty degeneration ratio and area of muscle/vertebral body were used to detect recovery of soft tissue.

**Results:**

The BDUA approach group was found to have significantly less intraoperative blood loss(*p* < 0.05) and postoperative drainage(p < 0.05) compared to conventional approach transforaminal lumbar interbody fusion group. Symptoms of spinal canal stenosis and nerve compression were significantly relieved postoperatively, as compared with the preoperative state. However, the opposite side had a lower rate of fatty degeneration (9.42 ± 3.17%) comparing to decompression side (11.68 ± 3.08%) (*P* < 0.05) six months after surgery in the BDUA group. While there were no significant differences (*P* > 0.05) in two sides of conventional transforaminal lumbar interbody fusion approach group six months after surgery.

**Conclusions:**

Bilateral decompression via unilateral approach (BDUA) is able to reduce the intraoperative and postoperative body fluid loss in the elderly. The opposite side of decompression in BDUA shows less fatty degeneration in 6 months, which indicates better recovery of the soft tissue of the aging patients.

## Background

Lumbar degenerative diseases are the most common spinal diseases in the aging population and are increasing in worldwide; among these diseases, lumbar degenerative disc disease is especially common. Between 2000 and 2009, 380,305 patients were diagnosed and underwent surgery, and the number of cases increased 2.4-fold in the United States [1]. The multifidus muscles, located on either side of the spinous processes, play an important role in stabilizing the joints within the spine [2]. Recent research has shown that among the paraspinal muscles, the multifidus muscle is associated with facet joint osteoarthritis, spondylolisthesis, and disc narrowing [3]. Imaging indicates that these diseases cause a decrease in muscle size and radiographic density, and an increase in fat deposits [4]. Recent research has shown that degeneration of the paraspinal muscles, especially the left muscle, are correlated with age [5]. The paraspinal muscles may also be replaced with fat in people with lumbar degenerative disease [6], and this replacement may be aggravated postoperatively [7]. In patients undergoing posterior lumbar interbody fusions, smaller area of the paraspinal muscles were associated with less fusion time [8]. In lumbar intervertebral disc surgery, bilateral decompression via a unilateral approach [9] (BDUA) has better results in terms of reducing the operation time, blood loss and other complications. However, no studies of prognosis, especially the recovery of the soft tissue, have reported using BDUA in an elderly population, such as in those over the age of 65. In addition, few studies have investigated postoperative multifidus muscle changes, particularly fatty degeneration. This study retrospectively analyzed 61 patients who received lumbar fusion between January 2016 and April 2018, and compared BDUA and conventional approach transforaminal lumbar interbody fusion.

## Methods

This retrospective case-control study was performed analyzing 61 aging patients with lumbar degenerative disease.

### Patient data

The inclusion criteria were as follows: 1. Single-segment degenerative disc herniation and spinal canal stenosis with neurological symptoms. 2. Age over 65 years. 3. More than one radiographic examination(X-ray, computed tomography (CT), Magnetic Resonance Imaging.

(MRI) or Diffusion tensor imaging (DTI))confirming nerve root compression. 4. Good general condition: blood pressure after intervention < 160 mmHg systolic and < 100 mmHg diastolic [10]; intraoperative blood glucose levels < 10 mmol/l (The Society for Ambulatory Anesthesia) [11]. Cardiopulmonary function, assessed by the anesthesiologist, is able to tolerate general anesthesia.

The exclusion criteria were as follows: 1. More than one segmental disc herniation. 2. Lumbago and no clear nerve root symptoms. 3. Advanced age (over 95 years). 4. Tumors. 5. Serious postoperative complications.6. morbid obesity. 7. Systemic disease or ane insufficiency.

Lumbar disc herniation was combined with stenosis in L4/5 in 47 patients, L3/4 in 3 patients and L5/S1 in 11 patients. Group A received bilateral decompression via a unilateral approach surgery, and group B received conventional transforaminal lumbar interbody fusion approach. All patients underwent lumbar X-ray, three-dimensional CT, and magnetic resonance imaging (MRI) before surgery. After surgery, all patients were followed up for 26.2 months, with a range of 20–36 months.

### Surgical methods

#### Bilateral decompression via unilateral approach (BDUA) group

**Fig. 1:**
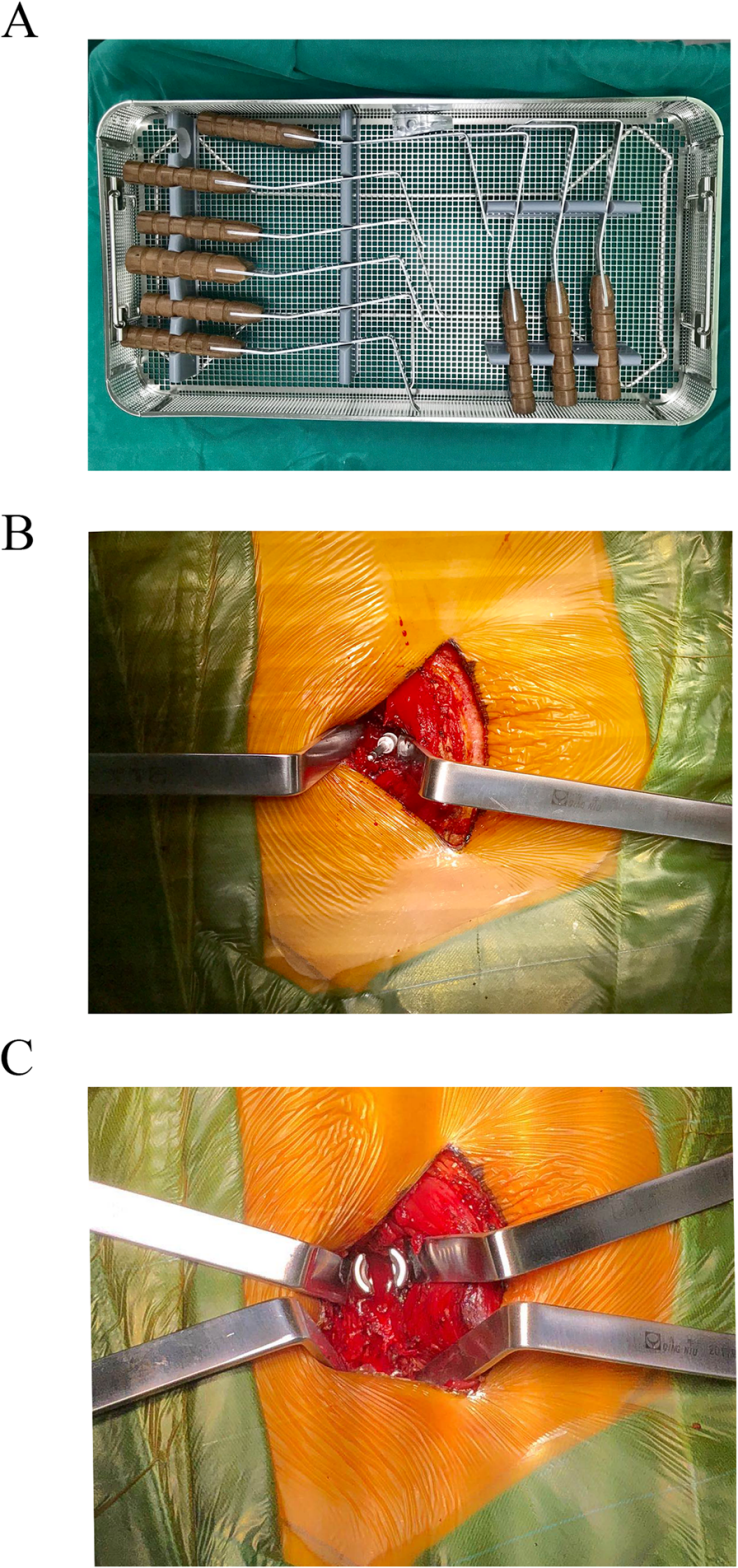
The exposure of the pedicle entry point through the Wiltse’s approach. **a**: The self-designed mini-retractor **b**&**c**: Intraoperative usage of the self-designed mini-retractor and the exposion of pedicle entry point

**Fig. 2:**
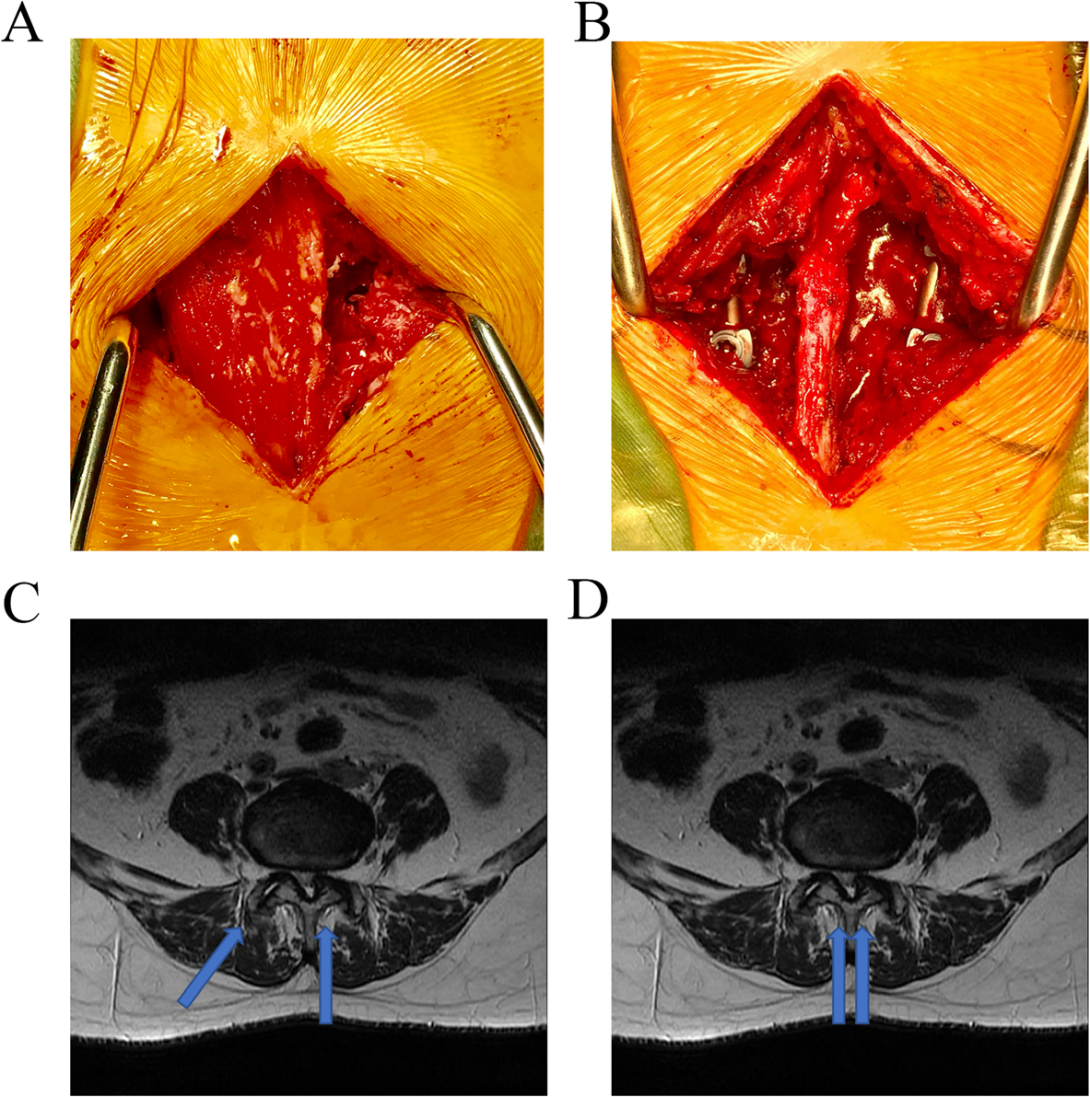
Schematic diagram and intraoperative photographs of the lumbar fusion operation. **a**&**c**: Intraoperative photographs(**a**) and schematic diagram(**c**) of bilateral decompression via unilateral approach; **b**&**d**:Intraoperative photographs(**b**) and schematic diagram(**d**) of conventional approach transforaminal lumbar interbody fusion

Each patient was placed in prone position and intubated under general anesthesia. A paravertebral incision was made in the lesion intervertebral space. The paravertebral muscle space was obtusely separated, With the help of mini-retractor designed by ourselves [12], multifidus and the longissimus muscles were separated and the pedicle entry point was exposed clearly (Fig. 1a,b,c). The pedicle screw was inserted into the target vertebra, and the articular process was removed with bone biting forceps. The vertebral plate after c-arm X-ray fluoroscopy confirmed that the reduction was satisfactory. During this process, the nerve root and dural sac were protected. Then the inferior facet and approximately 1/3 of the superior facet of symptomatic side were removed, the spinal canal was exposed, and the upper and lower laminar margins were removed depending on the specific conditions of spinal stenosis. Then the ipsilateral ligamentum flavum was completely removed. The contralateral view were obtained by tilting the operating table. Resection of the contralateral junction of lamina with the spinous process was performed in order to expanded spinal canal. At this point, the contralateral ligamentum flavum was excised. The soft tissue and osteophytes of the contralateral subarticular zone was excised to decompress the contralateral nerve root. Meanwhile, the protruded nucleus was removed, the intervertebral space was opened, and the cartilage of the vertebral endplate was removed for use in the bone graft fusion. The extracted articular process and lamina were used for granular packing in the intervertebral space, and the cancellous bone was compressed and placed into the intervertebral fusion cage. Wiltse’s approach was used to implant a contralateral pedicle screw. Finally, the incision was sutured after a negative pressure flow tube was placed. Fig. 2a,c shows an intraoperative photographs and schematic diagram of this surgical approach [13].

#### Control group: conventional approach transforaminal lumbar interbody fusion

Each patient was placed in prone position and intubated under general anesthesia. A standard midline incision and subperiosteal exposure is made out to the tips of the transverse processes and the longissimus and multifidus are separated from the posterolateral gutter. The pedicle screws were placed into the upper and lower vertebral bodies. The spinous process and bilateral lamina and ligaments were removed with bone biting forceps, whereas the ligamentum flavum and the medial edge of the articular process were removed according to the specific conditions of the disease. The nerve root canal and lateral crypt were expanded, and the dural sac and nerve root were protected intraoperatively. Then the annulus fibrosus was cut open, the nucleus pulposus was removed, upper and lower cartilage endplates were removed, and autologous bone particles were implanted between the vertebral bodies. The dural sac and nerve roots were then explored. Finally, the incision was sutured after a negative pressure flow tube was placed. Figure 2b, d shows a schematic diagram and intraoperative photographs of this surgical approach.

#### Postoperative management

All patients in the two groups had the drainage tube removed within 72 h and rested in bed for 3 days. Then, they were allowed to ambulate with the assistance of a lumbar brace within at least the next six weeks. All patients were followed up every three months, and X-ray, CT scans were reviewed. Besides,MRI were followed up in 3 and 6 months.

#### Clinical and radiological assessment

**Fig. 3:**
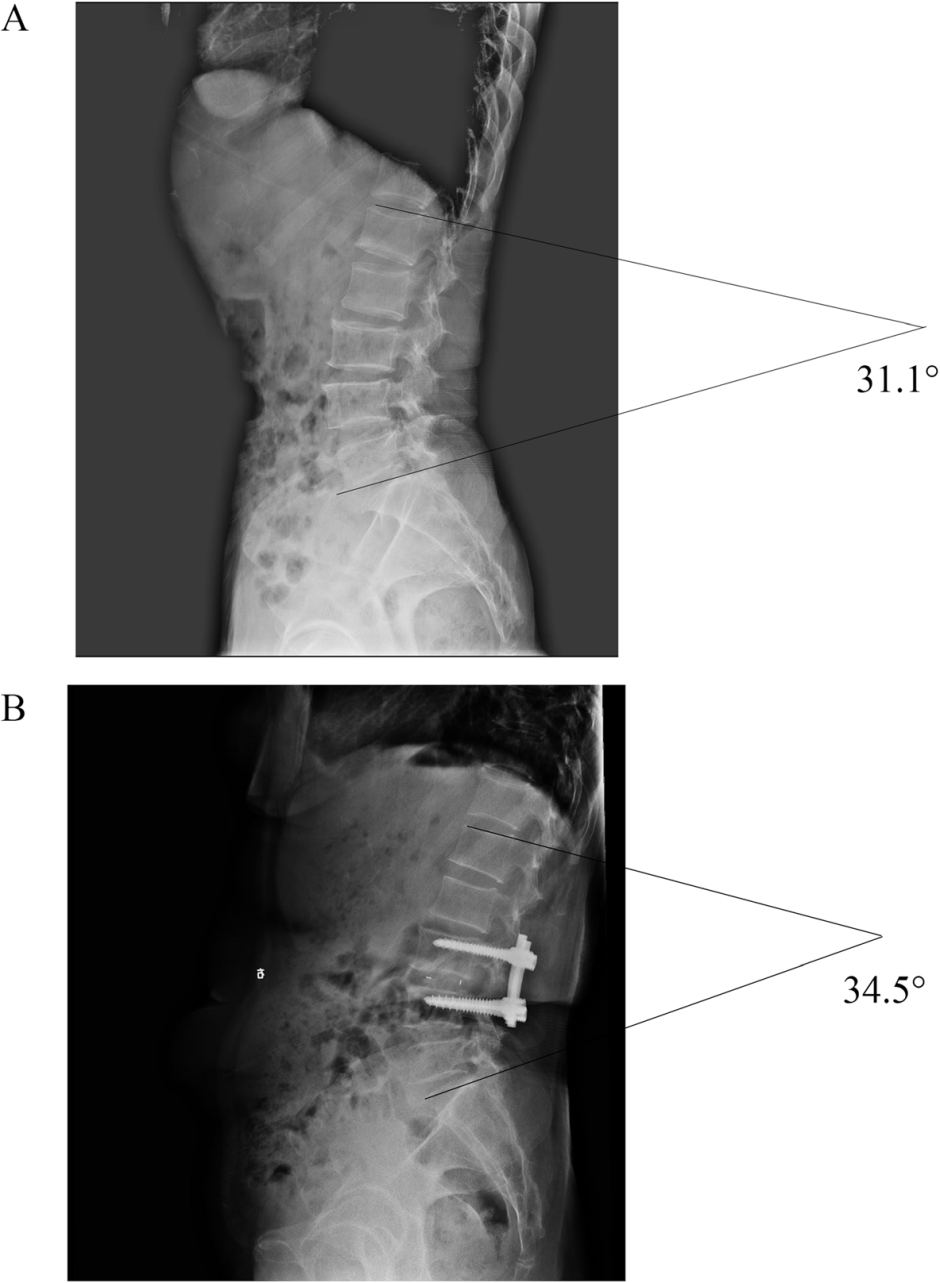
Mesurement of the L1-S1 lumbar lordosis. **a**: preoperative lumbar lordosis **b**: postoperativeb lumbar lordosis

**Fig. 4:**
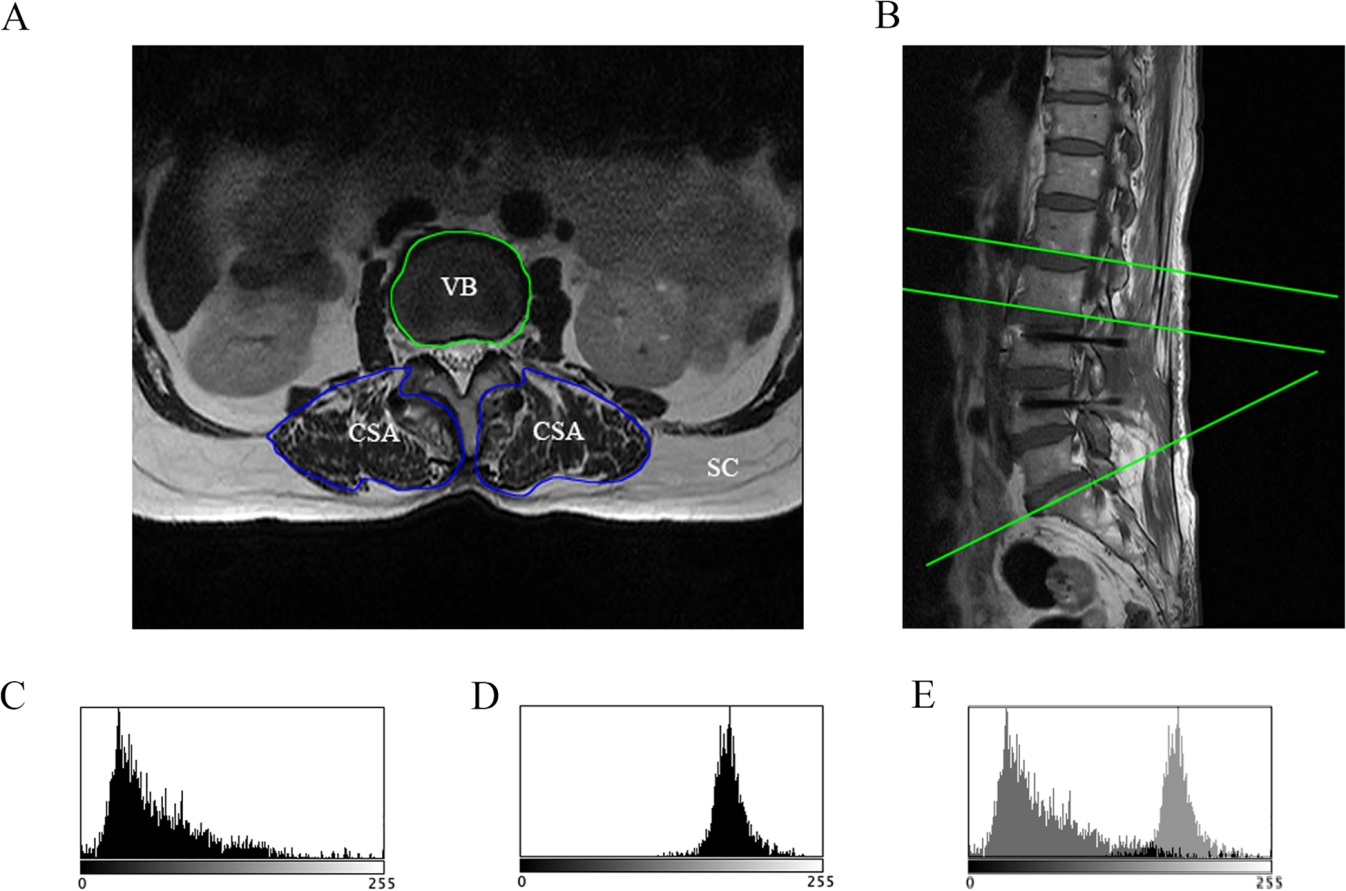
Mesurement of the multifidus muscle: **a**: T2-weighted axial slices of the lumbar spine **b**: the selection of the slices; **c**&**d**: The gray-scale range of the signal intensity histogram was measured within the cross-sectional area (**c**) and subcutaneous fat (**d**); **e**,: The grayscale value of the cross-sectional area region overlap with the subcutaneous fat region

All patients were assessed with Japanese Orthopedic Association (JOA), Visual Analogue Scale (VAS) and Oswestry Disability Index (ODI) scores before and after surgery. In addition, all patients were followed up every 3 months after surgery with X-ray, CT of the lumbar spine. MRI were followed up in 3 and 6 months. X-ray and CT were used to calculate the lumbar spine fusion rate through Lee’s radiographic criteria [14, 15]. X-ray was also used to measure L1-S1 lumbar lordosis of the standing position [16] (Fig. 3a, b). As shown in Fig. 4a,b, MRI was used to detect the fatty degeneration and muscle/vertebral body ratio [17]. VB represents the vertebral body size, CSA represents the cross-sectional area, and SC indicates subcutaneous fat. The calculation of the muscle/vertebral body ratio is also based on the cross-sectional area (CSA) and the vertebral body size (VB). The cross-sectional areas of the vertebral body and paraspinal muscles were outlined and measured by authors using Image J software (National Institutes of Health, MD, USA). Due to the cross-sectional area of the vertebral body would hardly change, the ratio of muscle/vertebral body can reflect the atrophy of paravertebral muscle. The gray-scale range of the CSA and SC areas was also analyzed in Image J software, as shown in Fig. 4c, d. The grayscale value of the CSA region overlap with the SC region (Fig. 4e) was used as an index of fatty degeneration of the multifidus muscle. In addition, the CSA/VB ratio indicated the degree of multifidus muscle atrophy. The surrounding layers of the lumbar fusion area were chosen to avoid metal interference. In order to unify the standard, the upper edge layer (inferior vertebral endplate) of the intervertebral disc in the upper segment of the fusion segment were selcected for the measurement.

### Statistical analysis

All data were analyzed in SPSS 22.0 software (IBM Corporation, NY, USA) and are presented as mean ± standard deviation. Differences between groups were tested by unpaired t test or Man-Whitney U test. Categorical variables were compared via chi-square test or Fisher exact test. Paired t test was used to compare affected side and opposite side within groups. A *P*-value of < 0.05 was considered statistically significant.

## Results

### Perioperative conditions

All 61 patients received the BDUA approach or conventional approach transforaminal lumbar interbody fusion under general anesthesia. All operations were performed successfully without any injury to the nerve root. After the operation, no serious complications such as deep infection, nerve injury, cerebrospinal fluid leakage, deep venous thrombosis or pulmonary infection occurred. The vacuum drainage was removed within three days postoperatively, and all patients were discharged three to four days after the operation. Symptoms of spinal canal stenosis and nerve compression were significantly relieved postoperatively, as compared with the preoperative state.

### Clinical and radiological results

The general data are shown in Table 1, including sex, age, BMI, high blood pressure, diabetes and the segment of the herniated disc. These factors were not significantly different (*P* > 0.05), thus excluding the influence of general factors on the results.

**Table 1:** Descriptive data and disease characteristics of patients

Variable	BDUA	Control	P-Value	Significance
Sex			0.3004^*^	ns
Male	16	20		
Female	15	10		
Age	72.26 ± 3.43	71.33 ± 3.41	0.2956	ns
65–70	6	10		
70–75	14	14		
over 75	11	6		
BMI	24.85 ± 2.93	24.87 ± 2.10	0.9875	ns
High blood pressure	11	6	0.2546^*^	ns
diabetes	4	5	0.7315^*^	ns
Segment of herniated disc			0.8068^*^	ns
L3/4	1	2		
L4/5	24	23		
L5/S1	6	5		

Note: Data shown as number or mean ± standard deviation. P < 0.05, significant difference

*χ^2^ test. Otherwise, independent samples t test

BMI, Body Mass Index

The average operation time for the BDUA group was 2.61 ± 0.78 h, whereas that of the conventional approach group was 2.85 ± 0.68 h. The operation times were not significantly different (P > 0.05). However, the BDUA group had the advantage of significantly less(*P* < 0.01) intraoperative blood loss (153.9 ± 102.9 ml) compared to the conventional approach group (251.7 ± 156.1 ml). In addition, wound drainage was significantly reduced(*P* < 0.0001) in the BDUA group (178.4 ± 86.7 ml), compared with the conventional approach group (359.8 ± 179.2 ml). The lengths of hospital stays between two groups has no significant difference (Table 2). Clinical outcomes are shown in Table 3, including the VAS, ODI and JOA scores. Most of the scores between two groups were not significantly different. However, the VAS score of the BDUA group three months after operation was 1.33 ± 0.48, whereas that of the control group was 3.45 ± 0.43. This result suggests that patients in the BDUA group had less pain in the early stages after surgery. However, at the last follow-up time, both groups showed significant pain relief. As shown Table 4, the fusion rate and lumbar lordosis between the groups were not significantly different (*P* > 0.05). MRI was performed to detect recovery of soft tissue (Table 5). We chose the surrounding layers to avoid metal interference. Both the fatty degeneration ratio and area of muscle/vertebral body were not significantly different (P > 0.05) preoperatively. However, the affected side had a higher rate of fatty degeneration six months after surgery in the BDUA group, thus indicating differences in postoperative recovery.

**Table 2:** Perioperative Factors and Postoperative Outcomes

Variable	BDUA	Control	P-Value	Significance
Operation time	2.61 ± 0.78	2.85 ± 0.68	0.2132	ns
Intraoperative blood loss	153.9 ± 102.9	251.7 ± 156.1	0.0059	**
Drainage	178.4 ± 86.7	359.8 ± 179.2	< 0.0001	****
Days of hospital stay	10.35 ± 3.49	12.07 ± 3.59	0.0642	ns
Complications cases	3	2	0.94^*^	ns
Side of fusion				
Both	0	30		
Left side	13	0		
Right side	18	0		

Note: Data shown as number or mean ± standard deviation. P < 0.05, significant difference

*χ^2^ test. Otherwise, independent samples t test

**Table 3:** The VAS,ODI and JOA scores of patients at different time points

Variable		BDUA	Control	P-Value	Significance
VAS scores	post-surgery	3.45 ± 0.89	3.07 ± 1.15	0.1464	ns
	3 months	1.33 ± 0.48	3.45 ± 0.43	< 0.0001	****
	Last time	1.25 ± 0.51	1.40 ± 0.62	0.3345	ns
ODI scores	post-surgery	43.48 ± 19.30	40.52 ± 15.53	0.4158	ns
	3 months	9.74 ± 6.71	10.33 ± 6.54	0.7287	ns
	Last time				
JOA scores	post-surgery	15.97 ± 5.12	17.13 ± 4.12	0.3507	ns
	3 months	24.90 ± 2.33	25.17 ± 1.82	0.6252	ns
	Last time	27.87 ± 2.40	28.27 ± 1.82	0.2777	ns
3 months	JOA improvement ≥75%	13	11		
	JOA improvement =50–74%	12	12		
	JOA improvement =25–49%	5	5		
	JOA improvement ≤24%	1	2		
Last time	JOA improvement ≥75%	26	28		
	JOA improvement =50–74%	4	1		
	JOA improvement =25–49%	1	1		
	JOA improvement ≤24%	0	0		

Note: Data shown as number or mean ± standard deviation. P < 0.05, significant difference. Independent samples t test

VAS, visual analog scale; ODI, oswestry disability index; JOA, Japanese Orthopaedic Association

**Table 4:** Radiologic assessments of patients at postoperation and follow-up

Variable	BDUA	Control	P-value	Significance
Fusion rate (% of patients)			0.8814	ns
3 months	83.8	93.3		
6 months	96.8	100		
last time	100	100		
Lumbar lordosis				
3 months	44.79 ± 8.15	46.30 ± 8.32	0.4797	ns
Last time	48.60 ± 7.61	51.50 ± 3.35	0.0606	ns

Note: Data shown as number or mean ± standard deviation. P < 0.05, significant difference

Independent samples t test

**Table 5:** Fatty degeneration ratio and lumbar muscularity of the paraspinal muscles of two groups using MRI

Variable	BDUA			Control			p-value	Significance
Fatty degeneration ratio %	preoperative	6 month	Change (%)	preoperative	6 month	Change (%)		
Affected side	7.96 ± 2.86	11.68 ± 3.08	2.69 ± 2.24	8.59 ± 3.77	11.38 ± 4.60	2.79 ± 4.57	0.9601	ns
opposite side	8.93 ± 3.48	9.42 ± 3.17	1.46 ± 2.80	8.89 ± 3.23	12.97 ± 4.58	4.09 ± 5.05	0.0143	*
p-value	0.2893	0.006		0.7472	0.1845			
Significance	ns	**		ns	ns			
Area of muscle/vertebral body %								
Affected side	137.9 ± 37.2	142.3 ± 45.1	4.5 ± 7.9	135.7 ± 16.1	149.9 ± 44.7	1.09 ± 11.4	0.1781	ns
Opposite side	149.4 ± 36.0	150.9 ± 39.9	1.5 ± 16.1	136.8 ± 37.3	145.7 ± 36.8	−4.11 ± 13.7	0.1039	ns
p-value	0.1487	0.3536		0.1685	0.3502			
Significance	ns	ns		ns	ns			

Note: Data shown as number or mean ± standard deviation. P < 0.05, significant difference

Independent samples t test

## Discussion

Age is the most significant factor in lumbar degenerative disease, in both the osseous structures and soft tissues. In osseous structures, the vertebral height and disc height are influenced the most. However, the relationship between age and lumbar disc height remains controversial. Khan [5] has found that disc heights, especially those of the L3 segment and L2/L3 disc, are influenced by aging, whereas Bach, by using CT scans, has found no significant difference between middle-aged and elderly people. Variation in disc height is determined much more by sex than by age. However, recent research has clearly indicated that aging is related to the loss of lumbar curvature [18], fatty infiltration in the lumbar paravertebral muscles [19], and loss of extensor muscle strength [20]. With increasing age, the lumbar muscle fat content changes [21] and causes degeneration of the lumbar spine. Yanik also found a significantly higher fatty degeneration in the paravertebral muscle in patients with low back pain by using the chemical shift MRI [22]. In addition, the occurrence of low back pain after prolonged bed-rest can lead to the atrophy of the paravertebral muscle [23]. However,the paravertebral muscles, especially the multifidus muscles, are influenced the most during the operation [24]. In this study we found that fatty degeneration occurred in the surrounding muscles after fusion surgery. It is reported that due to the loss of tendon adhesion to bone, a decrease in the length and number, myofibrinolysis and degeneration would happen in the muscle bars. Adipose tissue would accumulate in the muscle bundle, outside and inside the muscle [25]. Although we did not find significant postoperative atrophy of paravertebral muscle, BDUA surgery may cause less fatty degeneration due to the less destruction of muscle structure. Hildebrand found that increased severity of fat infiltration in the lumbar multifidus muscles correlated significantly with decreased range of motion of lumbar flexion [26]. The less muscle damage on the BDUA side, may also be helpful for postoperative recovery of motion of lumbar flexion. Thus, protecting the paravertebral muscles from the fatty degeneration may be a favorable option to improve the prognosis of aging patients.

TLIF, the most common approach in lumbar fusion surgery, was first proposed by Harms in 1982 [27]. All degenerative diseases, including degenerative disc disease, disc herniation and spinal canal stenosis, are indications for the TLIF approach. However, TLIF may lead to significant paraspinal muscle injury because of the incision of the lumbar multifidus [28]. Therefore, we used the intermuscular space between the paraspinal muscles. BDUA, first proposed by Young [29], decreases pressure on the side with greater symptoms (including the joints, joint resection, and enlarged nerve root canal and vertebral canal) and involves fusion through intervertebral bone grafting. The treatment limits damage to the spinal rear structure and maximizes retention of structures and ligaments of the posterior components. In addition, the operation reduces the postoperative incidence of low back pain and maintains the stability of the spine. On the milder side, we used the Wiltse approach to avoid injury from muscle dissection. This approach, first reported by Wiltse [30], uses the clearance between the multifidus and latissimus muscles to access the vertebral lamina. Because of the clearance involves connective tissues and avoids vessels and nerves, this approach limits the possibility of vascular injury and neurological impairment [31]. After the operation, the lumen can close by itself without drainage; therefore, none of our patients had any drainage on that side. Patients receiving the Wiltse approach had less postoperative drainage. In addition, the multifidus muscles can assist in the patient’s lateral position, and the integrity of the multifidus muscles can reduce the postoperative recovery times of patients.

TILF and PLIF, the two common surgical approaches in lumbar fusion surgery, have been modified, and MIS-TLIF minimally invasive surgical methods have been proposed [32, 33]. MIS-TLIF may be efficient in the treatment of one-level lumbar stenosis [34], especially in obese patients [35]. However, MIS-TLIF is not suitable for degenerative disease with severe spinal stenosis, and grade II or higher spondylolisthesis, PLIF and TLIF cannot be substituted in some patients. BDUA has the advantages of reducing paraspinal muscle injury, operation time,operation expense, blood loss and postoperative bed time; therefore, it is preferred for elderly patients. This approach is also suitable for more than one segment. However, BDUA may not be suitable for patients with a herniated disc on both sides.

There are still several limitations in our study: The follow-up time was too short, with the area of muscle/vertebral body and the fatty degeneration ratio was only calculated at 6 months, so the long-term follow-up still remains to be done. The gray value was used to calculate the fat infiltration ratio. However, the gray value of edema or inflammation in the paraspinal muscle would have similar to the gray value of fat. Edema or inflammation is reported occurred mainly in the first week postoperatively. So, we chose the MRI of 6 months, the period that fat degeneration will be the most obvious, to avoid the interference of the edema or inflammatory response [36]. No obvious edema or inflammation were found in the follow-up, but the slight edema or inflammatory cannot be excluded. Besides,the effect of fatty degeneration on early postoperative activities were not clearly followed up,and early postoperative activities are helpful in reducing postoperative complications such as pneumonia, lower limb thrombosis. No obvious postoperative complications occurred in the patients of this study yet. So, more cases are needed to figure out the correlation between less fatty degeneration in the BDUA group and postoperative complications.

In conclusion, Bilateral decompression via unilateral approach (BDUA) has the advantage in reducing the intraoperative and postoperative body fluid loss in the elderly and the opposite side of decompression in BDUA shows less fatty degeneration in 6 months. Whereas a long-term advantage could not be shown compared to conventional approach transforaminal lumbar interbody fusion.

## Data Availability

The data which analyzed during the study are stored in our hospital system and are available from the corresponding author on reasonable request.

## Abbreviations

CT: computed tomography
MRI: magnetic resonance imaging
VAS: visual analogue scale
ODI: Oswestry Disability Index
BDUA: bilateral decompression via unilateral approach
TILF: transforaminal lumbar interbody fusion
VB: vertebral body
CSA: cross-sectional areal
SC: subcutaneous fat
DTI: Diffusion tensor imaging
